# Medical image classification by incorporating clinical variables and learned features

**DOI:** 10.1098/rsos.241222

**Published:** 2025-03-12

**Authors:** Jiahui Liu, Xiaohao Cai, Mahesan Niranjan

**Affiliations:** ^1^ School of Electronics and Computer Science, University of Southampton, Southampton, UK

**Keywords:** medical imaging, classification, discriminant analysis, clinical variables, class activation map

## Abstract

Medical image classification plays an important role in medical imaging. In this work, we present a novel approach to enhance deep learning models in medical image classification by incorporating clinical variables without overwhelming the information. Unlike most existing deep neural network models that only consider single-pixel information, our method captures a more comprehensive view. Our method contains two main steps and is effective in tackling the extra challenge raised by the scarcity of medical data. Firstly, we employ a pre-trained deep neural network served as a feature extractor to capture meaningful image features. Then, an exquisite discriminant analysis is applied to reduce the dimensionality of these features, ensuring that the low number of features remains optimized for the classification task and striking a balance with the clinical variables information. We also develop a way of obtaining class activation maps for our approach in visualizing models’ focus on specific regions within the low-dimensional feature space. Thorough experimental results demonstrate improvements of our proposed method over state-of-the-art methods for tuberculosis and dermatology issues for example. Furthermore, a comprehensive comparison with a popular dimensionality reduction technique (principal component analysis) is also conducted.

## 1. Introduction

The integration of deep learning techniques with medical imaging modalities has become widespread in current research and clinical practice. This proliferation of machine learning and deep learning in the healthcare field is driven by their ability to successfully automate tasks, thereby alleviating the high cognitive workload of busy physicians [1,2]. In particular, the escalating workload demands in radiological imaging examinations imply that an average radiologist must interpret an image within a mere 3−4 s, working for 8 h a day. Such a workload contributes to fatigue and burnout, significantly increasing the error rate, which is an urgent concern to address in the context of patient lives. With the rapid advancement of deep learning technologies, an increasing number of endeavours have recently highlighted the widely discussed foundation models in the fields like computer vision and medical imaging [3,4]. This has led to a growing trend of effortlessly adapting these models to a wide range of downstream applications. Characterized by their vast scale on various datasets, foundation models have demonstrated remarkable capabilities in generating meaningful representations across multiple domains [5,6].

The field of modern medicine heavily depends on the integration of diverse data sources, such as imaging pixel data, structured laboratory data, unstructured narrative data and occasionally audio or observational reports, to inform clinical practice [7]. The outcome of diagnostic decisions is hard to be independently determined based on a single image. As it is known that radiologists generally express a desire for additional clinical information when interpreting images, this information significantly influences their reporting. In a survey conducted among radiologists, an overwhelming majority (87%) indicated that clinical information plays a substantial role in their interpretation process [8].

The accurate interpretation of imaging data, including radiology, pathology, ophthalmology and dermatology, relies heavily on the knowledge of clinical context. In this regard, the integration of relevant clinical and patient information assumes a crucial role in guiding the interpretation process, providing valuable insights for clinical decision-making and informing optimal patient care [9,10]. Early deep learning models in medical image analysis primarily focused on using pixel data as a single modality for input, without incorporating other clinical information as observed in actual medical practice (e.g. [11–13]). While those approaches have still contributed to the success of deep/machine learning in the healthcare field, they ultimately constrain their clinical translation. Other works, such as [14–17], employ pre-trained models for medical imaging without extensive fine-tuning, demonstrating that pre-trained models alone may offer valuable insights especially in the limited medical data scenario.

Our interest in this work lies in incorporating clinical contexts/variables, such as patient information, patient history, prior diagnoses and laboratory values, into the fusion of image features. This approach aims to simulate the diagnostic process carried out by radiologists in clinical practice. Note that, owing to the uniqueness of medical images and factors such as information confidentiality or the labour-intensive nature of data annotation, the available dataset size is often limited and therefore the medical image classification problem itself here is intrinsically challenging.

One primary benefit of employing deep learning in different fields is its capacity to autonomously extract features, eliminating the need for laborious manual feature engineering. Nevertheless, a corresponding drawback is the substantial volume of parameters needed by deep neural network’s multiple layers, a factor that can amplify the risk of overfitting to new and unseen data. One approach to address this challenge involves introducing additional training samples. However, this often becomes impractical in dealing with medical datasets owing to the expenses associated with conducting physical examinations and clinical tests on patients. In such cases, we believe reducing the dimensions of those high-dimensional representations could bring up better performance. Existing methods, such as pooling layers or simply passing the data through neural networks, however, may not effectively capture the discriminative nature of the data and be interpretable.

**Figure 1.**
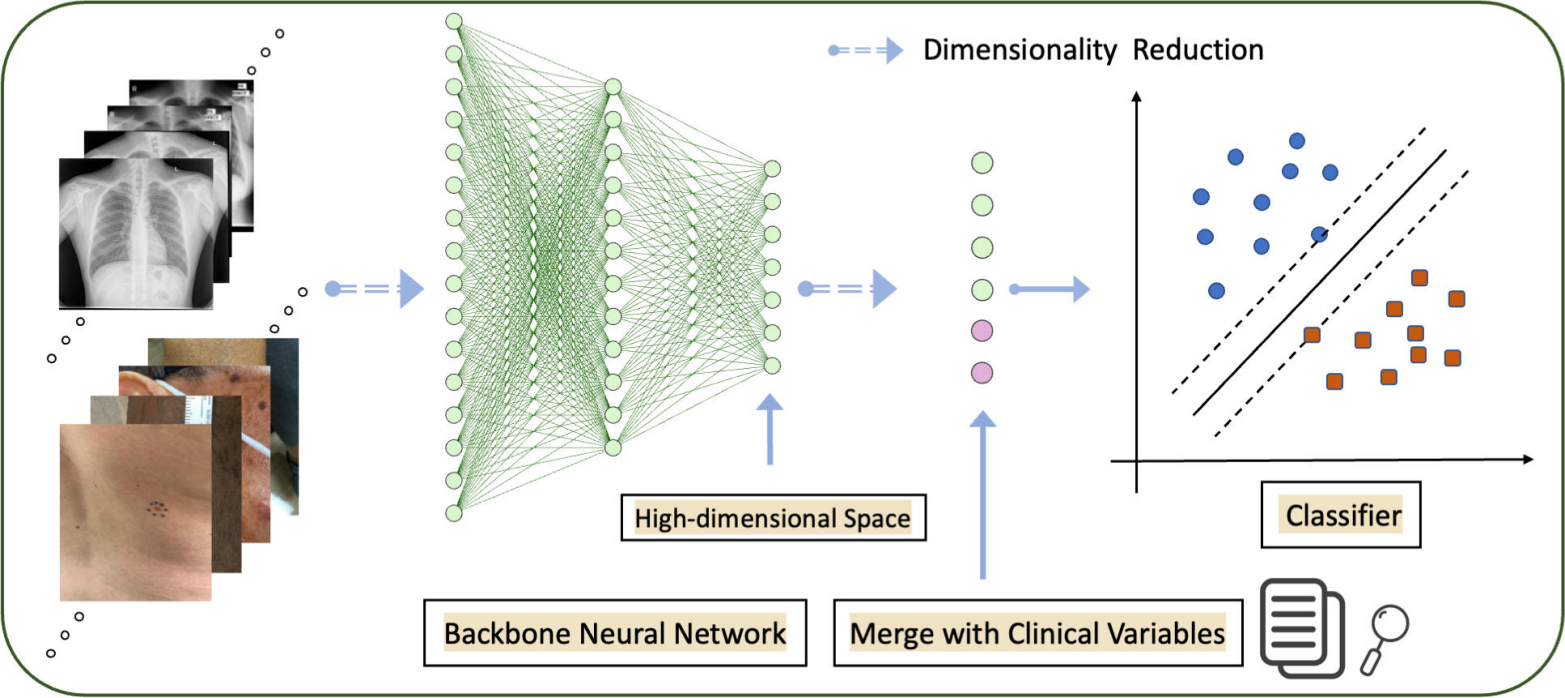
Schematic diagram of the proposed method for medical image classification by fusing extracted features with clinical variables. From left to right: a pre-trained deep neural network (e.g. ResNet18/ViT) is exploited to extract features (e.g. with 512/4096 dimensions) from input medical images, followed by subspace representations of the extracted features by dimensionality reduction using DA. The reduced features are then merged with clinical variables (which could also be embedded in a different space) and fed into a classifier (e.g. support vector machine (SVM) or a fully connected layer) for final prediction.

Our approach in this work incorporates clinical variables for medical image classification by using pre-trained deep neural networks and discriminant dimensionality reduction techniques. Specifically, we first map each medical image into a fixed-dimensional feature space, where the dimensions, denoted as M, are determined by the number of neurons in the penultimate fully connected layer of the network. Typically, popular architectures have dimensions of 1024 or 2048 for this layer. This leads us to a scenario where the number of available data items, denoted as N, is comparable with or even smaller than the dimension of the feature space, necessitating the use of techniques for dimensionality reduction. While previous works, such as [18], have used principal component analysis (PCA) for this purpose, we apply discriminant analysis (DA) [19] as a technique for dimensionality reduction, in that it effectively preserves discriminative information in the low-dimensional representations, enabling better separation of different categories of image data, especially in classification problems. Simultaneously, we find that this method helps localize the images within a subspace and reduce noise, while also convenient to incorporate clinical information as a foundation for subsequent analysis, preventing the overwhelming of these clinical variables with the features extracted by pre-trained networks. In detail, after obtaining the dimension-reduced features by DA, the clinical variables will no longer be overwhelmed by the high-dimensional feature space. We then merge the clinical variables with the dimension-reduced features and employ a classifier to make the final decision. The schematic diagram of our method is given in figure 1. Moreover, we also develop an innovative class activation map (CAM) for our method to visually represent the reduced features that accentuate regions pertinent to disease classification. This represents another distinctive contribution that has hitherto not been explored. Thorough experimental results are conducted to demonstrate the great performance of our proposed method over state-of-the-art (SOTA) methods in different disease classifications for example tuberculosis and dermatology. We also make a comprehensive comparison with the popular dimensionality reduction technique, i.e. PCA [20], further enforcing the superior performance of our proposed method. The code of our proposed method is available at: https://gitfront.io/r/Rachel/mGKvf81UJVr9/Incorporating-Clinical-Variables.

The remainder of this paper is organized as follows. In §2, we provide comprehensive details of related work including DA. Our proposed method is introduced in §3. Section 4 presents the main outcomes obtained from our experimental work. Lastly, §5 concludes the paper by summarizing our findings and offering further insights.

## 2. Related work

### 2.1. Fusion of imaging features with clinical variables

Although imaging findings for instance on chest X-rays consistent with pneumonia generally have imaging features that can differentiate alternative diagnoses, they are non-specific. Accurate diagnosis requires the incorporation of clinical and laboratory data [7]. In other words, imaging findings suggestive of pneumonia on chest X-rays may be accurate in a patient with supporting clinical features such as fever and elevated white blood cell count, but for another patient without those supporting characteristics and laboratory values, similar imaging findings may instead represent other aetiologies such as atelectasis, pulmonary oedema or even lung cancer. Such examples are abundant in the medical field, where single-pixel-level images alone cannot adequately replicate clinical practice. Similar to their real-life counterparts, automated detection and classification systems that can effectively use both medical imaging data and metadata from electronic health records, such as patient demographics, previous diagnoses and laboratory values, may result in improved performance and increased clinical relevance of the models. This idea has already been explored in recent literature, where neural networks were applied to extract features and then the extracted imaging features were simply concatenated with clinical features [21,22].

Kharazmi *et al*. [21] employed a sparse auto-encoder to capture hidden characteristics and obtain image representations for addressing a classification problem related to basal cell carcinoma skin cancer. They further generated feature maps, which were subsequently condensed through a pooling layer to reduce dimensionality. Following the reduction in dimensionality, these features were integrated with patient profile information (including lesion location, size, elevation, patient age and gender) and fed into the softmax layer. However, untrained features do not yield satisfactory results, especially when relying solely on the pooling layer for dimensionality reduction without any label information. Moreover, owing to the lack of loss updates, using only the pooling layer may result in a loss of valuable information. Numerous studies in the literature have employed similar pooling-based methods for dimensionality reduction [23,24].

Yap *et al*. [22] also addressed a skin lesion classification problem, incorporating metadata such as age, gender and body location. They achieved this by directly passing the image information to ResNet50 without including the last layer. Subsequently, the extracted features were concatenated with a metadata feature vector. Finally, the combined feature representation was passed to the softmax layer. Owing to the extensive architecture of the ResNet50 model, the feature representation before incorporating metadata already possesses a dimensionality of 1024. Combining a small amount of metadata with this representation would only result in further information loss. Therefore, they claim an improvement of less than 1%, which is even not consistently achieved. Similarly, Spasov *et al*. [25] adopted a comparable approach with three-dimensional structural magnetic resonance imaging (MRI) data to address Alzheimer’s disease classification. They proposed a convolutional neural network (CNN) architecture, which takes structural MRI scans and clinical assessment (e.g. age, gender, ethnic and racial categories, and years in education) as input. These fusion methods have also been widely employed in the context of predicting short-term future disease activity in patients with early symptoms of multiple sclerosis [26]. In line with this, they proposed a CNN architecture specifically designed for this purpose, which effectively extracted latent lesion features from MRI scans. Additionally, the model incorporated user-defined MRI and clinical measurements (e.g. body mass index (BOD), brain parenchymal fraction (BPF), gender, cerebrum, optic nerve, cerebellum, brainstem, spinal cord, extended disability status scale (EDSS) and cistype) at the second fully connected layer, allowing for a comprehensive fusion of data sources and improving the accuracy of disease progression predictions.

As mentioned in an earlier section, our approach in contrast involves obtaining features from a pre-trained deep learning model and subsequently employing DA to reduce the high-dimensional features and then combine them with clinical variables. We below briefly recall the fundamental mathematical concepts proposed by Foley & Sammon [19] to support the dimensionality reduction technique segment.

### 2.2. Discriminant analysis

In our proposed medical image classification method, we use DA to project the original features onto a lower-dimensional space while preserving discriminatory information. This reduction of dimensionality is achieved by minimizing the intra-class scatter and maximizing the inter-class scatter. The widely held assumption is that features based on discrimination are more effective than those based on fitting or describing the data. However, the traditional method of obtaining discriminant vectors for binary classification can only produce one direction owing to the rank of inter-class scatter. Foley & Sammon [19] discovered a method that employs orthogonality between vectors to generate a set of optimal discriminant vectors for binary classification. More details see below.

Given N samples ${𝒚}_{i}=(y_{i1},y_{i2},\cdots \;,y_{iM}{)}^{⊤}\in {\mathbb{R}}^{M},1\leq i\leq N$, we form a data matrix $𝒀=({𝒚}_{1},{𝒚}_{2},\cdots \;,{𝒚}_{N}{)}^{⊤}\in {\mathbb{R}}^{N\times M}$, where M is the number of features of every sample. Suppose that these N samples belong to c different classes, namely ${𝚲}_{j}$, and their cardinality $|{𝚲}_{j}|=N_{j}$, 1≤j≤c. Let $\bar{𝒚}$ and ${\bar{𝒚}}_{j}$, respectively, be the mean of the whole samples and the samples in class j, i.e. $\bar{𝒚}=\frac{1}{N}\sum_{i=1}^{N}{𝒚}_{i}$, ${\bar{𝒚}}_{j}=\frac{1}{N_{j}}\sum_{𝒚\in {𝚲}_{j}}𝒚$, 1≤j≤c. For c=2, let ${𝑺}_{B}$ and ${𝑺}_{W}$ denote the inter- and intra-class scatters, respectively, i.e.:


(2.1) $$\begin{array}{lcr} & {𝑺}_{B}={𝒔}_{b}{𝒔}_{b}^{⊤},\;{𝑺}_{W}=\beta {𝑺}_{W}^{1}+(1-\beta ){𝑺}_{W}^{2}, & \end{array}$$

where ${𝒔}_{b}={\bar{𝒚}}_{1}-{\bar{𝒚}}_{2}$ and $\beta =\left(N_{2}-1)/(N_{1}+N_{2}-2\right)$.

In the binary classification scenario, different from the traditional method which only produces one projection direction, the method in [19] offers the potential to discover additional discriminant directions. The optimality of this method stems from its ability to determine a set of projection directions, denoted as ${𝒅}_{n}$, satisfying multiple constraints provided below.

The projection direction, denoted by $𝒅\in {\mathbb{R}}^{M}$, plays a crucial role in Fisher discriminant analysis. The Fisher criterion reads:


(2.2) $$\begin{array}{lcr} & R(𝒅)=\frac{{𝒅}^{⊤}{𝑺}_{B}𝒅}{{𝒅}^{⊤}{𝑺}_{W}𝒅}. & \end{array}$$

It is noteworthy that the function R(𝒅) is independent of the magnitude of the projection direction 𝒅. In the context of binary classification, the optimal discriminant direction ${𝒅}_{1}$ is obtained by maximizing the value of R(𝒅). Once this has been achieved, we can derive


(2.3) $$\begin{array}{lcr} & {𝒅}_{1}=\alpha_{1}{{𝑺}_{W}}^{-1}{𝒔}_{b}, & \end{array}$$

where $\alpha_{1}$ is the normalizing constant such that $\|{𝒅}_{1}{\|}_{2}=1$ (i.e., $\alpha_{1}^{2}=({𝒔}_{b}^{⊤}[{𝑺}_{W}^{-1}{]}^{2}{𝒔}_{b}{)}^{-1}$). The second discriminant direction ${𝒅}_{2}$ is required to maximize R(𝒅) given in equation (2.2), while being orthogonal to ${𝒅}_{1}$. This can be accomplished using the method of Lagrange multipliers, i.e.:


(2.4) $$\begin{array}{lcr} & R({𝒅}_{2})-\lambda [{𝒅}_{2}^{⊤}{𝒅}_{1}], & \end{array}$$

where λ is the Lagrange multiplier. We can then obtain


(2.5) $$\begin{array}{lcr} & {𝒅}_{2}=\alpha_{2}\left({𝑺}_{W}^{-1}-\frac{{𝒔}_{b}^{⊤}({{𝑺}_{W}}^{-1}{)}^{2}{𝒔}_{b}}{{𝒔}_{b}^{⊤}({{𝑺}_{W}}^{-1}{)}^{3}{𝒔}_{b}}({{𝑺}_{W}}^{-1}{)}^{2}\right){𝒔}_{b}, & \end{array}$$

where $\alpha_{2}$ is the normalizing constant such that $\|{𝒅}_{2}{\|}_{2}=1$.

The aforementioned procedure can be recursively expanded to any number of directions in the following manner. The n-th discriminant direction ${𝒅}_{n}$ must maximize R(𝒅) in equation (2.2) while also being orthogonal to ${𝒅}_{k},k=1,2,\cdots \;,n-1$. It can be shown that:


(2.6) $$\begin{array}{lcr} & {𝒅}_{n}=\alpha_{n}{{𝑺}_{W}}^{-1}\left\{{𝒔}_{b}-\left[{𝒅}_{1}\cdots {𝒅}_{n-1}\right]{𝑺}_{n-1}^{-1}\left[\begin{array}{c} 1/\alpha_{1}\\ 0\\ \vdots \;\\ 0 \end{array}\right]\right\}, & \end{array}$$

where $\alpha_{n}$ is the normalizing constant such that $\|{𝒅}_{n}{\|}_{2}=1$ and ${𝑺}_{n-1}\in {\mathbb{R}}^{\left(n-1)\times (n-1\right)}$ whose (i,j) entries are defined as


(2.7) $$\begin{array}{lcr} & {𝒅}_{i}^{⊤}{{𝑺}_{W}}^{-1}{𝒅}_{j},\;\;1\leq i,j\leq n-1. & \end{array}$$

Through the above procedure, we are able to obtain $\{{𝒅}_{k}{\}}_{k=1}^{n}$ without being constrained by the rank of ${𝑺}_{B}$. These discriminant directions can then be used to reduce the dimensionality of our original obtained features to the desired low-dimensional subspace. Subsequently, they can be integrated with clinical information to support downstream tasks like medical image classification.

## 3. Proposed method

In this section, we present our proposed method for medical image classification and provide a comprehensive description of the use of the developed CAM on dimension-reduced features for visualization.

### 3.1. Proposed framework

Figure 1 depicts a simplified diagram illustrating the framework of the proposed method. As shown in the diagram, the left side represents the medical image data, say $\{{𝑰}_{i}{\}}_{i=1}^{N}$, where ${𝑰}_{i}$ represents one input image. The images are fed into a pre-trained deep neural network say ϕ, which has been trained on natural images from the ImageNet dataset in our case here. This network extracts high-dimensional image features ${𝒚}_{i}$ from every image ${𝑰}_{i}$, i.e. ${𝒚}_{i}=\phi ({𝑰}_{i})$, without performing fine-tuning. Subsequently, these features undergo dimensionality reduction by using the projection matrix


(3.1) $$P=(d_{1},d_{2},\cdots ,d_{L}{)}^{⊤}\in R^{L\times M},\;L\ll M,$$

obtained by DA introduced in §2.2, and we then have a low-dimensional feature vector


(3.2) $$\begin{array}{lcr} & {\bar{𝒚}}_{i}=𝑷{𝒚}_{i}\in {\mathbb{R}}^{L}. & \end{array}$$

Let ${𝒗}_{i}\in {\mathbb{R}}^{L_{\mathrm{cv}}}$ represent the $L_{\mathrm{cv}}$ number of clinical variables corresponding to image ${𝑰}_{i}$. We then combine the low-dimensional feature vector ${\bar{𝒚}}_{i}$ with the clinical information ${𝒗}_{i}$ and form a new feature vector say ${𝒛}_{i}$ (more details of the fusion strategies are given below). The resulting low-dimensional feature vector ${𝒛}_{i}$ is finally processed with mature machine learning classifiers (like support vector machine (SVM) or a fully connected layer) to make the final prediction.

Given the high costs of physical examinations and clinical tests, acquiring numerous medical images is generally impractical. Consequently, training a large deep neural network from scratch conventionally proves ineffective. To cope with the data scarcity in the medical domain, we use pre-trained deep neural networks, such as ResNet18, VGGNet or ViT, as feature extractors. Specifically, the medical images are firstly projected onto a fixed-dimensional feature space, congruent with the number of neurons present in the penultimate fully connected layer of a network. As previously mentioned, in this high-dimensional space, it often leads to data sparsity, overfitting and/or high complexity, making it imperative to employ pertinent dimensionality reduction techniques.

#### 3.1.1 Feature fusion strategies

We propose the following two distinct strategies to explore the integration of the features extracted from pre-trained models with clinical variables.

Our *first strategy* is straightforward. The feature vector ${𝒛}_{i}$ is formed by concatenating the features ${\bar{𝒚}}_{i}$ that have undergone dimensionality reduction with clinical variables ${𝒗}_{i}$, i.e.:


(3.3) $$\begin{array}{lcr} & {𝒛}_{i}=({\bar{𝒚}}_{i}^{⊤},{𝒗}_{i}^{⊤}{)}^{⊤}\in {\mathbb{R}}^{L+L_{\mathrm{cv}}}. & \end{array}$$

The *second strategy* involves extracting features from clinical variables ${𝒗}_{i}$ using a foundation model like BERT [27] to generate embeddings firstly, and then applying DA for dimensionality reduction on the clinical variable embeddings. Let ${\bar{𝒗}}_{i}$ represent the dimension-reduced clinical variable embeddings of ${𝒗}_{i}$. The feature vector ${𝒛}_{i}$ is analogously formed by concatenation, i.e.:


(3.4) $$\begin{array}{lcr} & {𝒛}_{i}=({\bar{𝒚}}_{i}^{⊤},{\bar{𝒗}}_{i}^{⊤}{)}^{⊤}. & \end{array}$$

A key consideration in our work is the nature of the clinical variable texts, such as patient gender and skin tone, which are often repetitive and lack diversity. Consequently the use of, for example the BERT foundation model in this context may not significantly enhance the performance of our method. We hypothesize that for the scenarios of datasets with a richer and more varied range of textual information, employing foundation models like BERT for text embeddings in our second feature fusion strategy could benefit the performance of our method.

We below present the detailed description of our developed CAM for our approach in figure 1 in visualizing models’ focus on specific regions within the low-dimensional feature space. For simplicity, the first fusion strategy in equation (3.3) is used for description.

### 3.2. Developed class activation map

CAMs can help us understand the specific regions and features that a model focuses on during image classification tasks [28]. Traditional CAM generates attention maps associated with each class by multiplying the input of the global average pooling layer with the weights of the classification layer. These attention maps can be overlaid on the original image to highlight the regions relevant to specific classes. However, this standard CAM pipeline does not directly support the visualization on dimension-reduced features. Below, for pre-trained CNN models, we present our developed CAM specifically designed for visualization of the classification decision process on dimension-reduced features. Here a fully connected layer say $\phi^{*}$ is used as the classifier (instead of SVM) to find the weights.

Let $𝑭\in {\mathbb{R}}^{M\times H\times W}$ be the feature map produced by the last convolutional layer of the pre-trained network ϕ corresponding to a given test image 𝑰, where M,H and W represent the number of channels, height and width of the feature map. For instance, if using the pre-trained ResNet18 model, 𝑭 will have dimensions 512×7×7, dependent on the choice of the model. After flattening the channel-wise feature map 𝑭, then 𝑭 is changed to a matrix say $\hat{𝑭}={\mathbb{R}}^{M\times H\cdot W}$. Note that in this case for image 𝑰, the high-dimensional image feature vector 𝒚=ϕ(𝑰) is obtained by global average polling on the feature map 𝑭 channel by channel with M=512. The feature vector 𝒚 is then projected into an L-length low-dimensional feature vector say $\bar{𝒚}$ by the projection matrix 𝑷, i.e. $\bar{𝒚}=𝑷𝒚\in {\mathbb{R}}^{L}$.

Let $𝑾=({𝒘}_{1},\cdots \;,{𝒘}_{c})\in {\mathbb{R}}^{(L+L_{\mathrm{cv}})\times c}$ be the weight matrix of $\phi^{*}$. Note that c is the total number of classes of the classification problem, $L_{\mathrm{cv}}$ is the total number of the clinical variables and ${𝒘}_{j},1\leq j\leq c$ is the weights vector for class j prediction of classifier $\phi^{*}$. Given that $\phi^{*}$ is a fully connected layer, for the fused feature vector 𝒛 corresponding to image 𝑰, the class label prediction for image 𝑰 is given by


(3.5) $$\begin{array}{lcr} & 𝒖={𝑾}^{⊤}𝒛\in {\mathbb{R}}^{c}. & \end{array}$$

Assume class i is predicted for image 𝑰, i.e. the largest component of 𝒖 is its i-th entry. We use the weight vector ${𝒘}_{i}$ to form the CAM. Let ${\hat{𝒘}}_{i}$ be the first L components of ${𝒘}_{i}$. The weight vector ${𝒘}^{\prime }$ for feature map $\hat{𝑭}$ is generated by


(3.6) $$\begin{array}{lcr} & {𝒘}^{\prime }={𝑷}^{⊤}{\hat{𝒘}}_{i}\in {\mathbb{R}}^{M}. & \end{array}$$

The initial CAM result is formed by reshaping ${\hat{𝑭}}^{⊤}{𝒘}^{\prime }\in {\mathbb{R}}^{H\cdot W}$ to a matrix ${𝑹}^{\prime }\in {\mathbb{R}}^{H\times W}$. The final CAM result 𝑹 is achieved by resizing ${𝑹}^{\prime }$ to the size of the test image 𝑰 by interpolation. The whole procedure of generating the CAM for models with dimension-reduced features is summarized in algorithm 1. Analogously, algorithm 1 is also applied to generate CAM for other dimensionality reduction techniques like PCA.

Once we obtain the CAM 𝑹, it is overlaid onto the original image. This overlay effectively highlights the discriminative regions of the image pertaining to different classes. By adopting this approach to dimensionality reduction, we are able to perceive the spatial feature information of the feature map, consequently augmenting the interpretability of the visualization results.


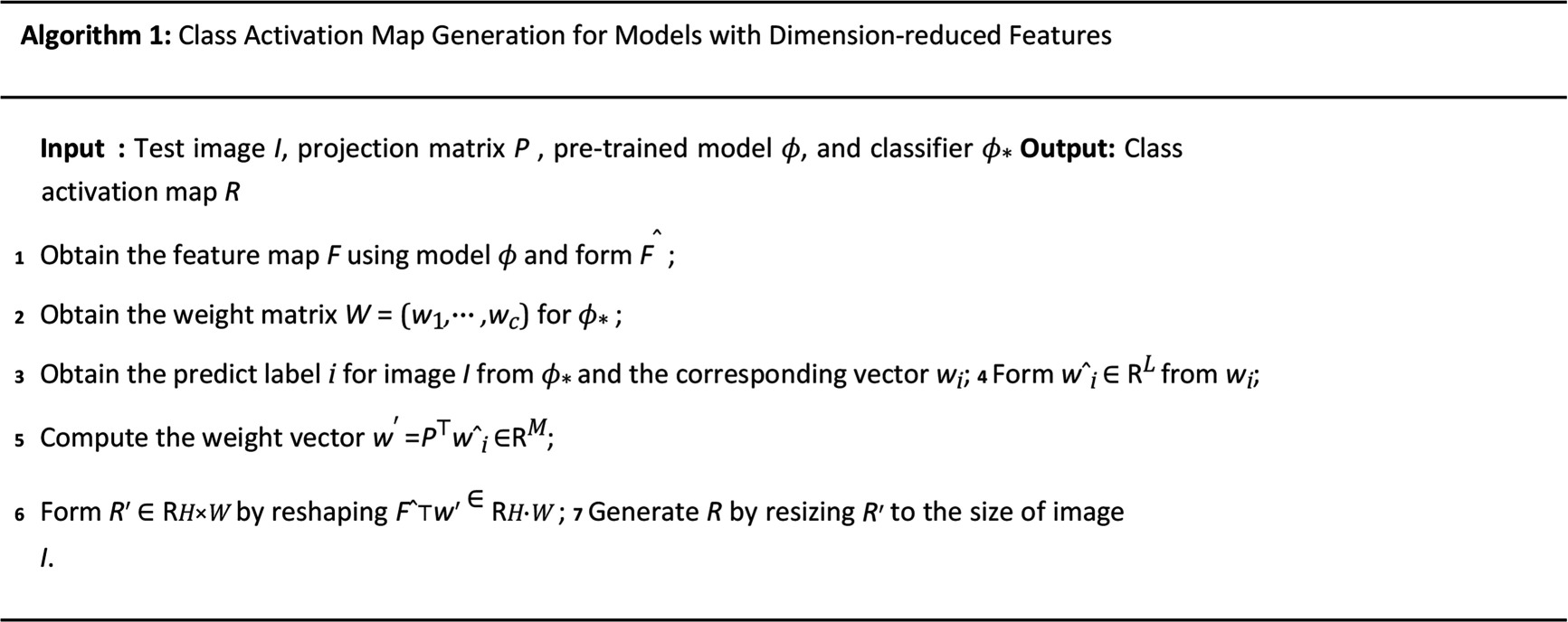


## 4. Experimental results

### 4.1. Experimental setting

In the experiments, to demonstrate the efficacy of our proposed method in addressing medical issues by considering clinical variables, four distinct datasets encompassing two different diseases are adopted (see below). We compare the performance of our method with SOTA techniques in [29,30] and commonly employed dimensionality reduction technique PCA, including different ways of using features. The performance of all the methods is evaluated by the metrics of accuracy (ACC) and the area under the curve (AUC).

To thoroughly test the performance of our method, we adopt various popular pre-trained deep neural networks, such as ResNet18, VGGNet16 and ViT, which have been trained on large natural image classification problems (i.e. ImageNet). These networks are used in the medical domain to obtain their respective representations. The foundation model BERT is also tested as our second feature fusion strategy with clinical variables for medical image classification. Unless particularly specified, our first feature fusion strategy is applied.

All the quantitative results are obtained under fivefold cross-validation, ensuring their reliability. The number of the discriminant directions L in our method is set to 10, i.e. the number of the original features is reduced to 10. The number of clinical variables considered in all the individual datasets is ≤3. All the experiments are conducted using Python 3.8. For the SVM classifier implementation, the scikit-learn library is used, with the Bayesian optimization process being facilitated by the bayes_opt library. Unless particularly specified, SVM is applied as the classifier in our framework.

### 4.2. Data

In our study, we concentrate on two distinct diseases, i.e. tuberculosis and dermatology, which both rely on metadata. The first disease tuberculosis poses a significant global health challenge. For tuberculosis-related investigations, we use two publicly available datasets, i.e. the Montgomery and Shenzhen datasets, published by the US National Institute of Health^1^. The second disease dermatology has led to significant morbidity and mortality worldwide, affecting millions of people and causing suffering even in developed countries [31]. For the study of dermatology, we use the publicly available dataset Diverse Dermatology Images (DDI)^2^ and PAD-UFES-20 dataset, which comprise diverse skin tones. Brief descriptions of these individual datasets together with our implementations are given below. Table 1 summarizes the number of images used and the associated clinical variables in the datasets. In particular, images from these datasets are resized for different pre-trained deep neural networks, i.e. the resized image sizes are 512×512, 224×224, and 384×384 for the pre-trained ResNet18, VGGNet16 and ViT, respectively.

**Table 1.** Data description in terms of the number of images and the clinical variables associated with each dataset.

data	number of images	clinical variables
Montgomery	138	age and gender
Shenzhen	662	age and gender
DDI	194	skin tone colour
PAD-UFES-20	384	tobacco consumption and age

- The Montgomery dataset [32] is a binary problem that was created by the National Library of Medicine in collaboration with the Department of Health and Human Services in Montgomery County, Maryland, USA. It consists of X-ray data collected as part of Montgomery County’s tuberculosis screening programme. In particular, it comprises 58 cases with tuberculosis manifestations and 80 normal cases, with images sized at 4020×4892 pixels. It also includes age and gender information as clinical variables. In our implementation, the dataset is balanced by random sampling.
- The Shenzhen dataset [32] represents another binary problem specifically related to tuberculosis and was collected in collaboration with Shenzhen No. 3 People’s Hospital, Guangdong Medical College, Shenzhen, China. This dataset comprises a collection of 662 frontal chest X-rays, among which 326 are classified as normal cases, while the remaining 336 exhibit manifestations of tuberculosis, including paediatric X-rays. The images in the dataset have varying sizes but are approximately 3000×3000 pixels. Additionally, it provides age and gender information as clinical variables for each case.
- The DDI dataset [30] was assembled retrospectively by reviewing histopathologically confirmed lesions diagnosed at Stanford Clinics between 2010 and 2020. It, comprising both dark and light skin tone images, was specifically designed for a binary problem. In our work, we exploit this dataset, using the images as well as additional clinical variables (i.e. skin tones). Notably, we incorporate 208 light skin tone images and 207 dark skin tone images in our analysis.
- PAD-UFES-20 [33] is a skin lesion dataset composed of patient data and clinical images. This collection was collated in conjunction with the dermatological and surgical support initiative at the Federal University of Espírito Santo. It encompasses six distinct skin lesions, i.e. basal cell carcinoma, squamous cell carcinoma (SCC), actinic keratosis (ACK), seborrheic keratosis, melanoma and nevus. Within the scope of our study, we consciously selected an equitable quantity of SCCs and ACKs, specifically 192 cases, while also considering variables such as alcohol and tobacco consumption, alongside the patients’ age, as clinical information.

### 4.3. Results of prediction performance

Table 2 shows the quantitative results of different methods using fivefold cross-validation with pre-trained ResNet18 in terms of the ACC and AUC metrics. The results of the existing SOTA methods are presented in the last column of table 2. The other six different approaches are following the framework in figure 1, with different ways of using features, i.e. original features obtained by using the pre-trained ResNet18 and dimension-reduced features obtained by using PCA and DA techniques individually, with and without incorporating clinical variables. Note that our proposed method is the one using DA with clinical variables (i.e. the penultimate column of table 2), achieving the best results among all the methods compared for all the datasets and the metrics. The results underscore the value of incorporating clinical variables in determining the presence of cancer, and the great performance of our method. It is understandable, as prior knowledge about patients’ baseline information is essential in the medical field for doctors making diagnoses and these individual details can indeed significantly influence the outcomes.

**Table 2.** Prediction performance comparison in terms of metrics ACC and AUC. (Four distinct datasets are used, with pre-trained ResNet18. Six different ways of using features following the framework in figure 1, with and without clinical variables, including the existing SOTA methods, are evaluated. ‘CV’ stands for ‘clinical variables’. Our method is the column of ‘DA + CV’, which performs the best for all cases.)

prediction performance across various feature utilization techniques								
data	metrics	original	original + CV	PCA	PCA + CV	DA	DA + CV	SOTA methods
Montgomery	ACC	0.82 ± 0.02	0.81 ± 0.05	0.71 ± 0.05	0.70 ± 0.06	0.82 ± 0.08	0.85 ± 0.08	0.79 [29]
	AUC	0.87 ± 0.05	0.87 ± 0.05	0.78 ± 0.02	0.78 ± 0.02	0.88 ± 0.05	0.89 ± 0.05	0.81 [29]
Shenzhen	ACC	0.87 ± 0.02	0.87 ± 0.02	0.85 ± 0.02	0.85 ± 0.02	0.87 ± 0.02	0.88 ± 0.03	0.84 [29]
	AUC	0.92 ± 0.01	0.92 ± 0.01	0.89 ± 0.01	0.90 ± 0.01	0.92 ± 0.01	0.93 ± 0.01	0.90 [29]
DDI	ACC	0.71 ± 0.02	0.72 ± 0.01	0.66 ± 0.03	0.68 ± 0.04	0.73 ± 0.01	0.74 ± 0.02	N/A
	AUC	0.74 ± 0.07	0.75 ± 0.06	0.70 ± 0.06	0.71 ± 0.07	0.75 ± 0.08	0.77 ± 0.08	0.72 ± 0.05 [30]
PAD-UFES-20	ACC	0.67 ± 0.01	0.66 ± 0.02	0.60 ± 0.02	0.61 ± 0.03	0.69 ± 0.02	0.70 ± 0.02	N/A
	AUC	0.71 ± 0.02	0.70 ± 0.04	0.61 ± 0.05	0.63 ± 0.06	0.71 ± 0.04	0.72 ± 0.02	N/A

Table 3 presents results in a similar manner of table 2 but using a fully connected layer as a classifier instead of the SVM classifier. Analogous performance is obtained as that in table 2, i.e. our proposed method again achieves the best results among all the methods compared for all the datasets and the metrics. It is also observed that the SVM classifier is slightly better than the simple fully connected layer, which is reasonable since the SVM classifier is optimized by the built-in library. It is worth highlighting that using a fully connected layer will assist us to form CAMs for feature visualization, results of which will be deferred in next subsection.

**Table 3.** Prediction performance comparison in terms of metrics ACC and AUC with the same settings as in table 2 except that a fully connected layer is used as the classifier instead of the SVM classifier. (Again, our method (i.e. the column of ‘DA+CV’) performs the best for all cases.)

prediction performance across various feature utilization techniques							
data	metrics	original	original + CV	PCA	PCA + CV	DA	DA + CV
Montgomery	ACC	0.72 ± 0.05	0.72 ± 0.06	0.65 ± 0.04	0.66 ± 0.04	0.76 ± 0.06	0.77 ± 0.07
	AUC	0.83 ± 0.05	0.84 ± 0.06	0.74 ± 0.03	0.76 ± 0.05	0.85 ± 0.06	0.86 ± 0.07
Shenzhen	ACC	0.84 ± 0.03	0.84 ± 0.04	0.82 ± 0.03	0.82 ± 0.03	0.86 ± 0.02	0.87 ± 0.03
	AUC	0.93 ± 0.02	0.93 ± 0.02	0.89 ± 0.03	0.89 ± 0.03	0.93 ± 0.01	0.93 ± 0.01
DDI	ACC	0.68 ± 0.05	0.68 ± 0.06	0.65 ± 0.06	0.66 ± 0.08	0.71 ± 0.05	0.72 ± 0.07
	AUC	0.72 ± 0.09	0.73 ± 0.10	0.69 ± 0.09	0.70 ± 0.11	0.72 ± 0.11	0.73 ± 0.11
PAD-UFES-20	ACC	0.67 ± 0.04	0.66 ± 0.06	0.61 ± 0.02	0.62 ± 0.02	0.66 ± 0.03	0.68 ± 0.02
	AUC	0.72 ± 0.04	0.70 ± 0.04	0.60 ± 0.06	0.59 ± 0.05	0.70 ± 0.03	0.72 ± 0.04

Table 4 presents further results in a similar manner of table 2, where the original features are extracted by using the pre-trained VGGNet16 and the widely popular ViT model separately. Note that the number of feature dimensions by different pre-trained models can be different, e.g. 4096 dimensions in the penultimate layer of the VGGNet16 compared with the 512 dimensions in ResNet18. Specifically, the results in the first and second columns of table 4 (and tables 2 and 3) are almost identical, indicating that directly incorporating high-dimensional features without dimensionality reduction along with clinical variables leads to the clinical variable information being overshadowed within the vast feature space. The results also demonstrate the necessity and usefulness of dimensionality reduction by DA against PCA which achieves poor results, as the original high-dimensional features contain redundancy information which may be misleading, with the number of dimensions exceeding even the number of samples. Again, our proposed method achieves the best results among all the methods compared for all the datasets and the metrics except for one outlier on the DDI dataset in terms of the AUC metric, demonstrating the great performance of our method and the significant improvement in predictive performance when incorporating clinical variables after dimensionality reduction by DA. It is also observed that among the pre-trained networks ResNet18, VGGNet16 and ViT, they each have better performance on some datasets, showing the diversity of the features they produce.

**Table 4.** Prediction performance comparison with the same settings as in table 2 but pre-trained VGGNet16 and ViT.

prediction performance across various feature utilization techniques							
data	metrics	original	original + CV	PCA	PCA + CV	DA	DA + CV
results with pre-trained VGGNet16							
Montgomery	ACC	0.76 ± 0.07	0.76 ± 0.07	0.69 ± 0.06	0.69 ± 0.06	0.81 ± 0.06	0.82 ± 0.06
	AUC	0.85 ± 0.06	0.85 ± 0.06	0.79 ± 0.06	0.79 ± 0.06	0.89 ± 0.04	0.90 ± 0.04
Shenzhen	ACC	0.86 ± 0.03	0.86 ± 0.03	0.83 ± 0.02	0.83 ± 0.02	0.87 ± 0.02	0.88 ± 0.02
	AUC	0.90 ± 0.02	0.90 ± 0.02	0.89 ± 0.02	0.89 ± 0.02	0.92 ± 0.01	0.92 ± 0.01
DDI	ACC	0.68 ± 0.03	0.68 ± 0.03	0.61 ± 0.04	0.61 ± 0.04	0.70 ± 0.07	0.71 ± 0.08
	AUC	0.74 ± 0.05	0.74 ± 0.05	0.61 ± 0.07	0.61 ± 0.07	0.73 ± 0.09	0.73 ± 0.08
PAD-UFES-20	ACC	0.63 ± 0.03	0.64 ± 0.04	0.62 ± 0.04	0.63 ± 0.03	0.67 ± 0.05	0.67 ± 0.04
	AUC	0.65 ± 0.05	0.65 ± 0.04	0.63 ± 0.06	0.64 ± 0.07	0.68 ± 0.08	0.69 ± 0.08
results with pre-trained ViT							
Montgomery	ACC	0.68 ± 0.05	0.68 ± 0.04	0.66 ± 0.08	0.66 ± 0.08	0.76 ± 0.04	0.77 ± 0.05
	AUC	0.77 ± 0.09	0.77 ± 0.09	0.67 ± 0.14	0.67 ± 0.13	0.80 ± 0.09	0.81 ± 0.09
Shenzhen	ACC	0.82 ± 0.02	0.82 ± 0.02	0.79 ± 0.01	0.79 ± 0.02	0.82 ± 0.01	0.82 ± 0.02
	AUC	0.87 ± 0.02	0.87 ± 0.02	0.87 ± 0.01	0.87 ± 0.01	0.86 ± 0.01	0.87 ± 0.01
DDI	ACC	0.76 ± 0.06	0.76 ± 0.06	0.70 ± 0.10	0.70 ± 0.10	0.78 ± 0.05	0.79 ± 0.05
	AUC	0.79 ± 0.08	0.79 ± 0.08	0.70 ± 0.14	0.70 ± 0.13	0.81 ± 0.09	0.83 ± 0.07
PAD-UFES-20	ACC	0.65 ± 0.04	0.66 ± 0.05	0.62 ± 0.02	0.64 ± 0.01	0.68 ± 0.04	0.70 ± 0.04
	AUC	0.67 ± 0.05	0.68 ± 0.04	0.66 ± 0.03	0.66 ± 0.02	0.70 ± 0.05	0.71 ± 0.05

Table 5 presents the results using different base models for feature extraction with the second feature fusion strategy (i.e. the foundation model BERT is used for clinical variable embeddings). It shows that the results using the second feature fusion strategy are not as good as using our first feature fusion strategy, but are better than the ones without incorporating the clinical variables (cf. tables 1–4), again demonstrating the importance of clinical variables in disease prediction. We hypothesize that as the complexity of clinical variables increases, the use of foundation models like BERT for feature embedding from text could become evident. This could be a significant aspect of ongoing research endeavours.

**Table 5.** Prediction performance comparison in terms of metrics ACC and AUC, with different pre-trained models and the second feature fusion strategy (i.e. the foundation model BERT is used for clinical variable embeddings).

prediction performance across various feature extraction models				
data	metrics	ResNet18+BERT	VGGNet16+BERT	ViT+BERT
Montgomery	ACC	0.85 ± 0.07	0.81 ± 0.07	0.75 ± 0.07
	AUC	0.89 ± 0.05	0.89 ± 0.05	0.80 ± 0.09
Shenzhen	ACC	0.88 ± 0.02	0.87 ± 0.02	0.75 ± 0.04
	AUC	0.93 ± 0.01	0.92 ± 0.03	0.80 ± 0.04
DDI	ACC	0.72 ± 0.07	0.69 ± 0.09	0.74 ± 0.07
	AUC	0.73 ± 0.12	0.71 ± 0.11	0.79 ± 0.10
PAD-UFES-20	ACC	0.67 ± 0.02	0.67 ± 0.04	0.67 ± 0.04
	AUC	0.70 ± 0.03	0.69 ± 0.08	0.69 ± 0.02

### 4.4. Results of class activation map visualization

Finally, we show the developed CAMs, which are capable of emphasizing regions of interest on dimension-reduced features, visually highlighting regions of the input image that play a significant role in classification prediction. This visualization could improve network comprehension and provide visual assistance when generating diagnostic reports for radiologists.

**Figure 2.**
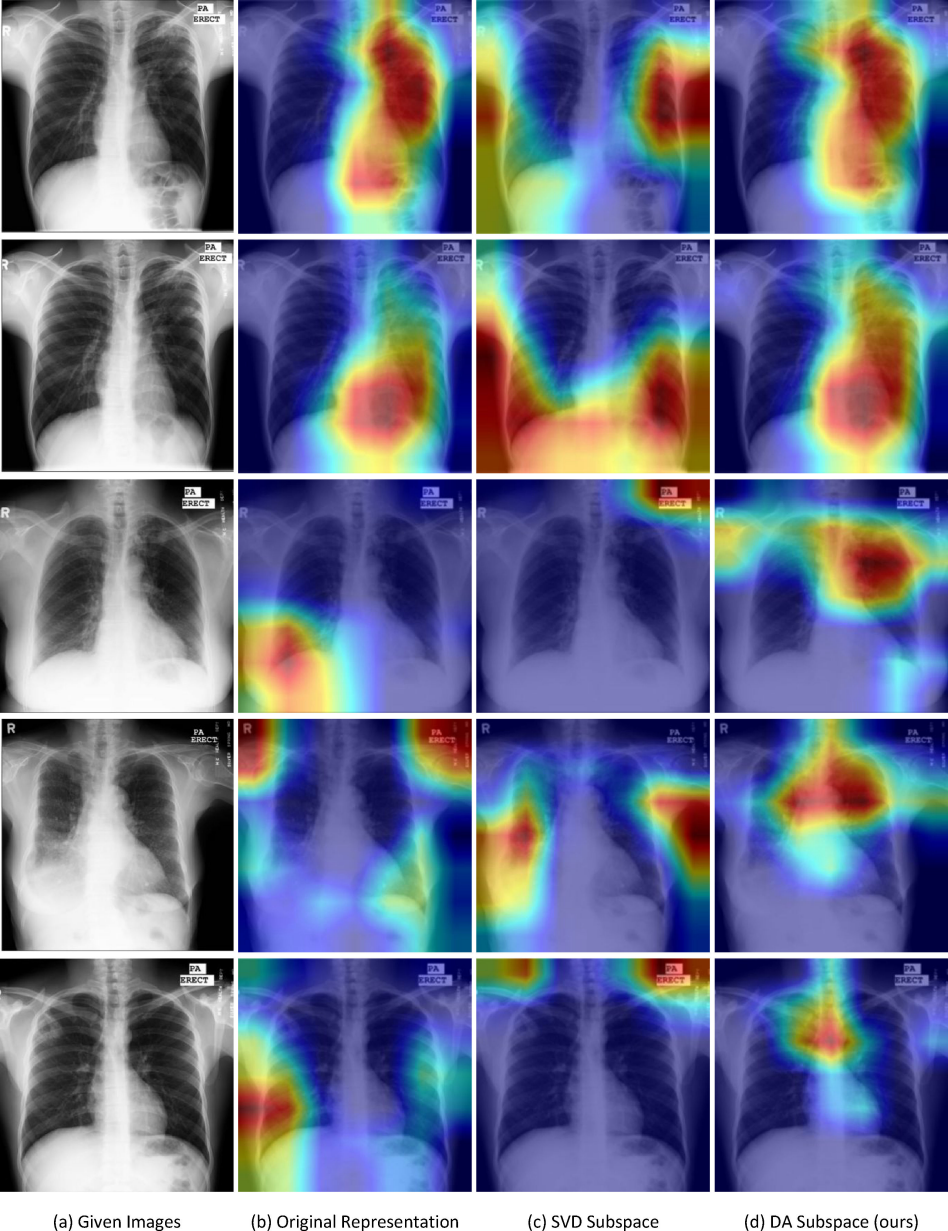
Visualization examples of CAMs on the Montgomery dataset. Column 1: given images; columns 2−4: CAMs regarding the original features, the dimension-reduced features by SVD and by DA, respectively. In particular, the first two rows illustrate instances where all the methods achieve correct classification prediction; the subsequent two rows display scenarios where only our method achieves correct classification prediction; and the final row highlights an example where all the methods make incorrect classification prediction.

Figure 2 presents some CAM examples on the Montgomery dataset, covering different cases like correct/incorrect classification predictions. For all the cases, we see that the CAMs of our proposed method can preserve superior localization (i.e. more centralized), compared with the ones obtained by using the original features and the dimension-reduced features by PCA. Moreover, the highlighted areas in the CAMs of our method are more focused on the chest area, whereas the focus of the CAMs related to the original features and the dimension-reduced features by PCA is quite random and deviates from the chest area (see the last three rows in figure 2).

**Figure 3.**
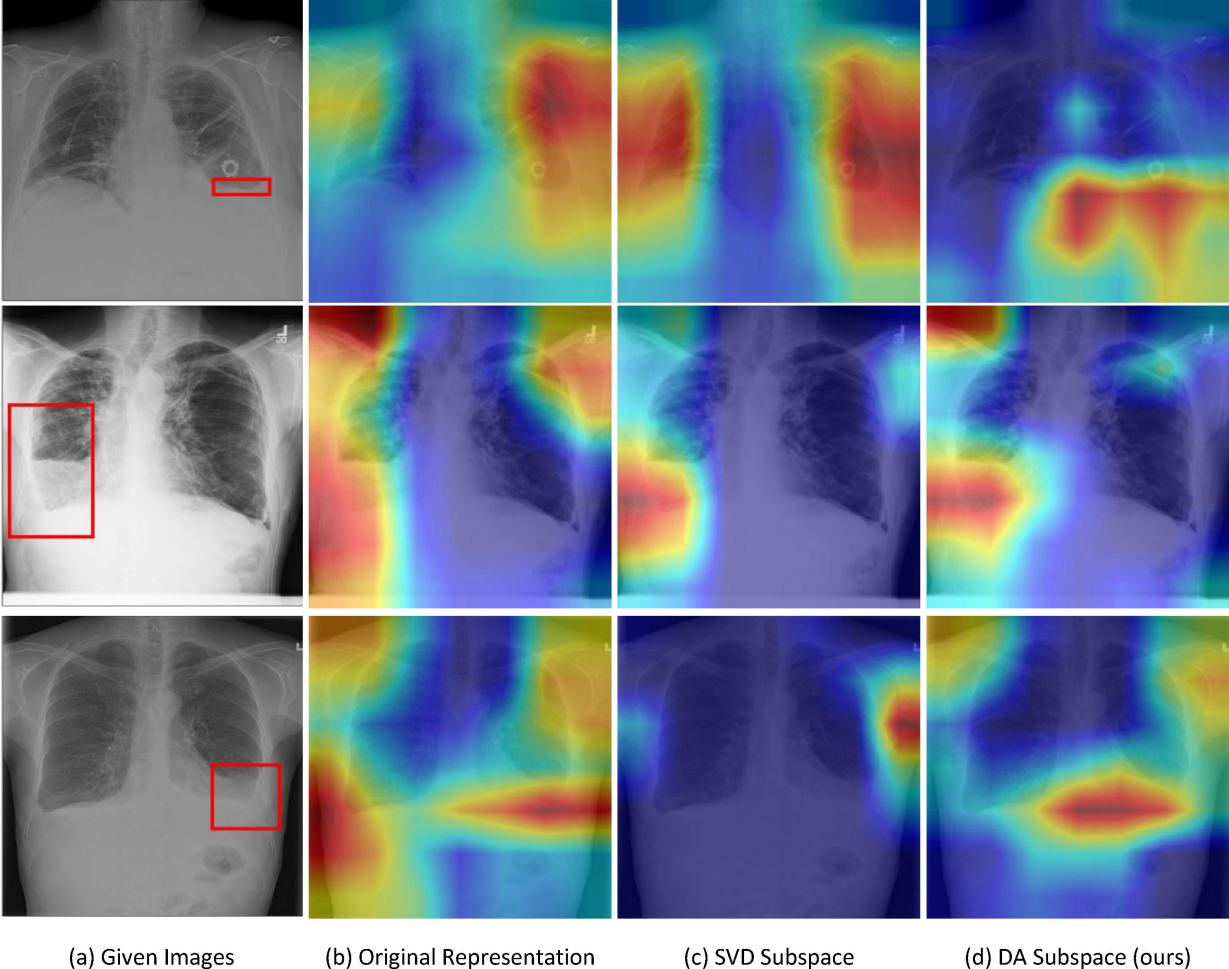
Visualization examples of CAMs on the ChestX-ray8 dataset with the inclusion of bounding box information regarding the areas of interest. All the methods achieve correct classification prediction for the given images.

To gain more insight into how the outcomes of our method align with the diagnoses of radiologists in treating patients, we integrate the ChestX-ray8 dataset [34], which contains a select group of pathological images accompanied by manually annotated bounding boxes indicating the areas of interest. Figure 3 illustrates the performance of different methods on effusion conditions, with instances where all the methods make correct classification prediction. It shows that only the CAMs of our proposed method highlight the areas that are more consistent with the manually annotated bounding boxes provided, demonstrating that our proposed method can effectively focus on regions relevant to radiologists’ assessments.

### 4.5. Discussion and limitation

We used pretrained deep neural networks in combination with DA for dimensionality reduction, allowing us to extract highly discriminative features that we integrate with clinical variables. This integration is designed to simulate the real diagnostic process by balancing image data with clinical metadata, ensuring neither overwhelms the other. Our primary goal is not to push marginal improvements in benchmark accuracy but to prioritize practical applicability in clinical diagnosis. While some approaches that use more complex neural network models may report higher accuracy, they often do so at the expense of lack of generalization, transparency and interpretability—trade-offs that are critical in medical settings. Moreover, the datasets we use are limited in terms of the richness of clinical variables, which constrains the full potential of our model. To address this, more clinically relevant data could be incorporated in future work to further validate and enhance the model’s performance. We remark that CAMs in our current approach do not directly visualize the influence of the clinical data, in that the clinical data is not linked to the pre-trained deep neural networks; in other words, it is not extracted by the pre-trained deep neural networks. The combination of image features (obtained by pre-trained deep neural networks and DA) and clinical variables in our framework can enhance decision-making and accuracy in a way that aligns with real-world diagnostic scenarios.

## 5. Conclusion

In this paper, we proposed a method for medical image classification by using the integration of clinical information with pixel-wise images using machine/deep learning techniques, yielding great performance in medical classification problems. Our empirical analysis mainly involved four medical datasets crossing two different diseases and various clinical variables. Our main findings are: (i) pertinent dimensionality reduction is crucial prior to combining clinical variables, demonstrated by the DA dimensionality reduction approach and our proposed feature fusion strategies for dimension-reduced features and clinical variables; (ii) DA outperforms well-known techniques such as PCA by a large margin owing to its ability of preserving more discriminatory information; (iii) the combination of clinical variables with pixel-wise images reflects real-world clinical practice and is proved to be beneficial for decision-making in the medical field; and (iv) the visualization through the developed CAMs further validates that our proposed method indeed focuses on regions aligned with that of the pathologist whereas other methods cannot. These findings contribute to the advancement of using machine/deep learning schemes in healthcare applications.

## Data Availability

All data used in this study are from publicly available datasets.

## Footnotes

^1^
https://openi.nlm.nih.gov/faqfaq-tb-coll.

^2^
https://stanfordaimi.azurewebsites.net/datasets/35866158-8196-48d8-87bf-50dca81df965.
